# Haitian women in New York City use global food plants for women’s health

**DOI:** 10.1186/s13002-024-00648-1

**Published:** 2024-01-12

**Authors:** Ella T. Vardeman, Edward J. Kennelly, Ina Vandebroek

**Affiliations:** 1 The Institute of Economic Botany, The New York Botanical Garden, 2900 Southern Boulevard, Bronx, NY 10458 USA; 2 PhD Program in Biology, The Graduate Center, City University of New York, 365 5th Ave, New York, NY 10016 USA; 3 Department of Biological Sciences, Lehman College, City University of New York, 250 Bedford Park Blvd W, Bronx, NY 10468 USA; 4 Department of Life Sciences and Caribbean Centre for Research in Bioscience (CCRIB), Faculty of Science and Technology, The University of the West Indies, Mona, Kingston 7, Jamaica

**Keywords:** Haiti, Women’s health, Urban ethnobotany, Cross-cultural comparisons, Ethnobotanical fieldwork, Traditional medicine

## Abstract

**Background:**

Despite the availability of mainstream biomedical healthcare in New York City (NYC), community-based ethnomedicine practices remain a low-cost, culturally relevant treatment for many immigrants. Previous urban ethnobotany research in NYC has established that several Caribbean communities continue using medicinal plants for women’s health after immigration. This study sought to address to what extent: (1) NYC Haitian women continue using medicinal plants for women’s health after migration; (2) their plants and the conditions treated were similar to those identified in an earlier survey with NYC immigrants from the Dominican Republic.

**Methods:**

Through an ethnobotanical survey, 100 Haitian women living in NYC and born in Haiti were interviewed about their knowledge of medicinal plants for women’s health conditions. Reported species were purchased based on local names in NYC Haitian stores and markets, vouchered, and identified.

**Results:**

Nearly all Haitian women (97%) reported using medicinal plants while living in Haiti. Most Haitian women continued using medicinal plants after coming to the USA (83%). The 14% decrease, although significant (*z* = 3.3; *p* = 0.001), was mainly due to logistical difficulties with sourcing plants after recent immigration. Popular medicinal plant species reported were primarily global food plants, re-emphasizing the intertwined food-medicine relationship in Caribbean diasporas. Comparison with data from NYC Dominicans identified childbirth and puerperium, gynecological infections, and vaginal cleansing as priority Haitian women’s health concerns treated with plants.

**Conclusion:**

Our findings support the global nature of Caribbean migrant plant pharmacopeia, predominantly centered around food plants and adapted to transnational urban settings. They underscore cultural diversity, dispelling the notion of one uniform traditional knowledge system labeled “Caribbean.” The importance of preventative medicine for women’s health, particularly the regular consumption of “healthy” foods or teas highlights the role food plants play in maintaining health without seeking treatment for a particular condition. Cross-cultural comparisons with other NYC Caribbean immigrants emphasize the importance of conducting ethnobotanical surveys to ground-truth plant use in the community. Such surveys can also identify culture-specific health priorities treated with these plants. Healthcare providers can leverage these insights to formulate culturally relevant and community-tailored healthcare strategies aligned with Haitian women’s health beliefs and needs.

**Supplementary Information:**

The online version contains supplementary material available at 10.1186/s13002-024-00648-1.

## Introduction

The Haitian diaspora is estimated to measure 1.7 million people globally, with the largest population of Haitians outside of Haiti, over 700,000 people, living in the USA [1]. Economic and political instability in Haiti has driven waves of immigration to the US, with peaks following the aftermath of the Haitian revolution (1791–1810), US occupation of Haiti (1915–1934), and during (1957–1986) and after (1986–1994) the Duvalier dictatorships [2]. In the past two decades, natural disasters and sociopolitical crises such as the 2010 earthquake and the political instability following the 2021 assassination of President Jovenel Moïse prompted US Homeland Security to grant Haitian migrants Temporary Protection Status [3, 4]. The Haitian diaspora in the US is primarily concentrated in Florida and New York City (NYC) [2, 5]. Haitians comprise one of the largest immigrant communities in NYC with an estimated over 80 thousand people [6].

As people migrate to new environments, they bring their ethnomedicinal traditions, including medicinal plants, with them [7]. Previous research has shown that the Haitian diaspora continues to use medicinal plants after migration [8–10]. However, until now there has not been an ethnobotanical survey focused on the Haitian community in NYC. The use of medicinal plants is not only an essential aspect of Haitian culture, but also has a deep history as a method to combat lack of access to biomedicine in Haiti [11]. As Haitians and other immigrants come to the USA, many experience new barriers to healthcare such as immigration status, lack of medical insurance, English proficiency, or sufficient income [12–14]. Haitian women experience particular barriers to biomedical care for their reproductive health both before and after migration, such as gendered familial duties and mistrust of the medical community, making the use of medicinal plants for women’s health even more important [15].

The existing Haitian ethnobotanical literature has been limited in scope compared to other Caribbean and Latin American countries [16]. For example, there are several papers on the ethnobiology of the Haitian zombie, someone who has become “undead” or comatose as the result of witchcraft [17–19]; however, the most comprehensive work focused on documenting plants used in Haiti was conducted as part of Traditional Medicine in the Islands (TRAMIL) in the late 1980s [20, 21]. This research was published decades ago and largely excluded plants used for women’s health by only listing plants used as abortifacients, emmenagogues, or for amenorrhea. Since the early twenty-first century, the literature on medicinal plants has expanded within the Haitian diaspora in the Caribbean, such as in Cuba and French Guiana, and in North America, such as Miami and Montreal [8, 10, 22–24]. In the USA, there has been some public health research focused on the medicinal plants for Haitian women’s health in Miami, but these surveys were not from an ethnobotanical perspective [25, 26].

Urban ethnobotany fieldwork with other Caribbean communities has demonstrated the rich and dynamic traditional medicine as an alternative healthcare system for women’s health conditions in NYC [27–29]. For example, Dominican immigrants integrated knowledge from other cultures and adapted their plant pharmacopeias to species more readily available in NYC [30]. Medicinal plants used to treat non-communicable diseases, such as women’s reproductive health issues, became even more culturally important after migration from the Dominican Republic (DR) [31]. Haiti and the DR are both located on the island of Hispaniola and share a similar flora [32]. After migration to NYC, there is also a shared flora available in *botánicas*, grocery stores, herbal markets, and public parks. A *botánica* is a Latino and Caribbean healing store that sells dried and/or fresh plants, and religious artifacts, such as candles, statues, good luck amulets and books. These stores may also offer spiritual guidance and consultations to customers. Cross-cultural comparisons between different immigrant communities can identify population-specific healthcare needs that can inform public policy and cultural sensitivity training for healthcare professionals [33, 34].

This paper aims to answer the following research questions: Do NYC Haitian women continue using medicinal plants for women’s health after migration? If so, what is the extent and depth of their medicinal plant knowledge? Do NYC Haitians use similar plants as NYC Dominicans for women’s health?

Therefore, based on the substantial plant knowledge brought to NYC by other related immigrant communities, we hypothesized that Haitians would have a thorough knowledge of medicinal plants as well as overlapping use of plant species and women’s health concerns with previously collected data from the NYC Dominican community. An exploratory analysis based on participant demographics, including age and migration history, was used to verify whether any of these variables correlated with medicinal plant knowledge.

## Methods

### Study area and informant consent

The primary dataset used in this analysis is based on original fieldwork carried out from September through October 2022 consisting of an ethnomedicine survey with the Haitian community in NYC. This survey focused specifically on medicinal plants used for women’s health conditions. The primary dataset was compared to a previously published ethnomedicinal survey with Dominicans living in NYC and the DR between 2005 and 2006 [30]. The latter dataset contained plant use data for 30 different common health conditions prevalent in the Dominican community, including seven symptoms and conditions related to reproductive health such as infertility, contraception and abortion, menstrual pain, vaginal discharge, labor and puerperium, and sexually transmitted infections, as well as additional symptoms reported, unprompted, by Dominican interview participants, such as vaginal cleansing and HIV/AIDS. We defined puerperium as the post-partum period after birth that is relevant to women’s health.

**Fig. 1:**
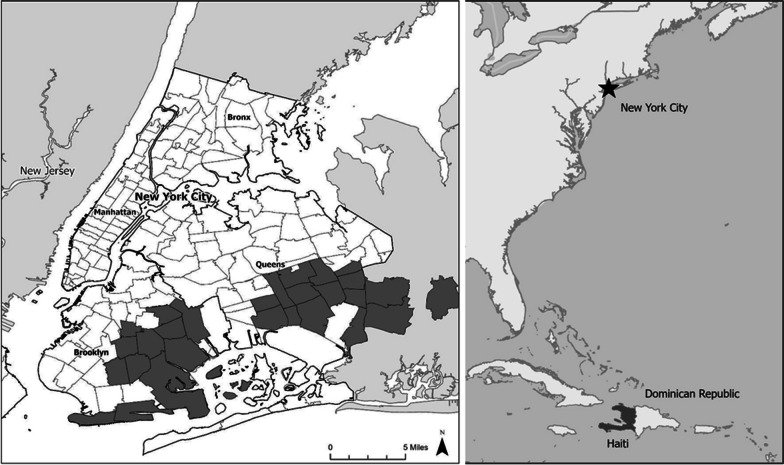
Study area (highlighted in dark gray) for NYC Haitian survey (left); Haiti (highlighted in dark gray) and Dominican Republic on the island of Hispaniola and NYC (star) are also shown on map (right)

Haitian interview participants met the following criteria: they were immigrants born in Haiti, 18 years old or older, had self-reported familiarity with medicinal plants, and consented to be interviewed. Participants were recruited through convenience and snowball sampling [35]. Specifically, we walked around in the Haitian neighborhoods of Flatbush in Brooklyn, also known as Little Haiti, and Jamaica in Queens conversing with people about the purpose of the study. We also visited a Haitian church, other Haitian community centers and businesses, and collaborated with Haitian Americans United for Progress whose staff members facilitated participant recruitment and provided translational aid where needed. In NYC, one hundred Haitian women were interviewed in Brooklyn and Queens (Fig. 1). The City University of New York (CUNY) Institutional Review Board (IRB) reviewed the study and all materials used (questionnaire and verbal consent forms in English) and granted permission for this study (IRB #2022-0107-Lehman).

Participants were lay people with a general knowledge of plants for women’s health. The age of participants ranged from 21 to 75 years and was 42 ± 15 years on average, whereas they had lived in the USA for 8 ± 12 years. All participants received an ID to maintain anonymity. The questionnaire was administered verbally in English and/or Haitian Creole and lasted about one hour or less, with the first author asking questions and writing down participant answers, either directly or with the help of a Haitian translator. The questionnaire included quantitative and qualitative components to assess plant knowledge for women’s health and questions focused on: (1) medicinal plant use before and after migration; (2) a list of 18 women’s health conditions where participants were asked to list remedies for each condition; (3) beliefs surrounding medicinal plant use for women’s health; and (4) demographic information. The list of women’s health conditions was developed based on data from a previous ethnobotanical survey with the Dominican community [30]. Participants were also asked for herbal remedies that they take regularly to improve their general reproductive health (women’s health preventatives). The questionnaire recorded plants used (and their forms of preparation and/or administration) by their common Creole names. At the end of the questionnaire, participants were asked to list any other relevant women’s health conditions not named as part of the survey that they had familiarity with treating with medicinal plants. To thank them for their time, participants received a 20 USD token of appreciation.

### Identification of medicinal plants

**Fig. 2:**
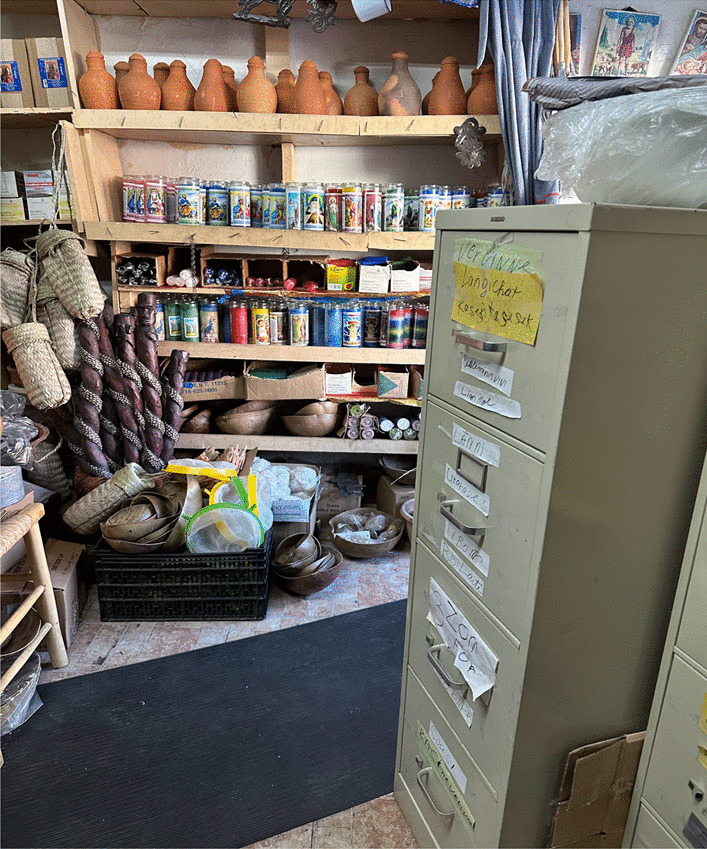
Haitian variety store in Little Haiti, Brooklyn

Reported plants were collected and vouchered by purchasing plant material in Haitian variety stores (cultural stores with Haitian products) and Haitian street vendors in Little Haiti (Fig. 2). In total, seven stores were visited, and staff were asked if they had plant material available based on common plant names. Specimens were then identified through the existing biocultural collection and botanical reference works at the New York Botanical Garden (NYBG). Voucher specimens were deposited at the William & Lynda Steere Herbarium (NY). The associated label data will be scrutinized by the NY collections managers to make informed decisions on the cultural information that should be publicly available online. In general, herbarium specimens are digitally available through the C. V. Starr Virtual Herbarium (http://sweetgum.nybg.org/science/vh/). If withheld information is needed for further research, that information can be made available through a data request to the authors and/or the NY Director. Scientific plant names and geographic distribution followed the Catalogue of Life (https://www.catalogueoflife.org), and plant family names followed APG IV (http://www.mobot.org/MOBOT/research/APweb/).

### Data collection and analysis

Interview data, stored in a secure Microsoft Access database, can only be accessed by project leaders. Databases were exported to Microsoft Excel for further analysis. Each record represents a plant–use report that includes the health condition, the common name of the plant used, and information on preparation and administration. Information on the reporting participant (including ID, age, and gender) for each plant–use report is also listed. Popular plant species are defined as those reported independently by three or more participants. Popular health conditions for which these plants are used are defined as those reported by five or more participants. Statistical analysis was performed in Microsoft Excel and PSPP 1.6.2. Raw data used for linear regression analysis were first subjected to square root transformation to enhance normality and equality of variances. *Z* tests were used to analyze significance for proportions based on one variable, and Chi-square analysis for goodness of fit was used to compare the number of participants reporting different women’s health conditions. Finally, the Jaccard similarity index (JSI) was used to compare the number of unique plant species between surveys [36].

## Results

### Importance of medicinal plants in Haitian culture before and after migration

Almost all Haitian participants interviewed in this study (97%) used medicinal plants in general (for any health condition) when they lived in Haiti (Table 1). After moving to NYC, most participants still use medicinal plants (83% *z* = 3.3; *p* = 0.001), although fewer than before migration. However, people said this was mainly due to logistical difficulties with sourcing plants after recent immigration (Table 1).

**Table 1:** Prevalence of medicinal plant use by Haitian women before and after migration to NYC (*n* = 100)

		Number of NYC Haitian women reporting
**1. Used medicinal plants while living in Haiti**		97
Reasons for not using medicinal plants:		
	Young when left Haiti	2
	Does not know about dosage	1
**2. Uses medicinal plants while living in NYC**		83
Reasons for not using medicinal plants:		
	Does not know where to buy plants or recently immigrated	9
	Does not use plants regularly	2
	Plants are not available in NYC	2
	Pregnant, or not currently taking plants for medical reasons	2
	Plants are not safe	1
	Plants are not needed in NYC	1

Participants indicated that there is an established system for sourcing Haitian medicinal plants in NYC. Most participants who used medicinal plants in NYC purchased these plants from Haitian-specific stores and vendors (59%), other members of the NYC Haitian community (13%), or directly from Haiti (31%) (Table 2).

**Table 2:** Sources of medicinal plants for NYC Haitians

	Number of NYC Haitian women who use medicinal plants reporting (*n* = 83)
NYC Haitian stores and vendors	49
Directly from Haiti	26
Family/friends/Haitian community in NYC	11
Local parks/gardens in NYC	4
Other US cities	3
Asian stores in NYC	2
Haitian communities in France and Canada	1
Church	1
*Botánicas*	1
No answer	22

Knowledge of medicinal plants for women’s health was not dependent on age, time spent living in Haiti, or time living in the USA. Older participants did not know significantly more plants than younger women. Participants who had moved away from Haiti at a young age or had lived many years in the USA did not show a statistical difference in medicinal plant knowledge from participants who had lived in Haiti most of their lives or had recently immigrated.

### Popular medicinal plants used by Haitian women for women’s health

In total, our ethnobotanical survey resulted in a list of 203 plant species used for Haitian women’s health in NYC. We reduced this list to 73 popular species that were independently reported by at least three survey participants (Additional files 1 and 2). Included in this list are food plants which are species primarily used for consumption and/or are common in either Haitian or Dominican cuisine and have a secondary role as medicines. These include vegetables, spices, Caribbean-specific staple foods, fruits, and condiments, such as *Cajanus cajan* (L.) Huth (*pwa kongo*, pigeon pea), *Chamissoa altissima* (Jacq.) Kunth (*lyann panye*, false chaff flower), *Mangifera indica* L. (mango), and *Syzygium aromaticum* (L.) Merr. & L.M.Perry (*jiwòf*, clove), and excludes others, such as *Cymbopogon citratus* (DC.) Stapf (*sitwonel*, lemongrass) which holds significance in Asian cuisine but is not a focal element in Caribbean cooking. The latter are called here non-food medicines (hereafter “non-foods”).

**Fig. 3:**
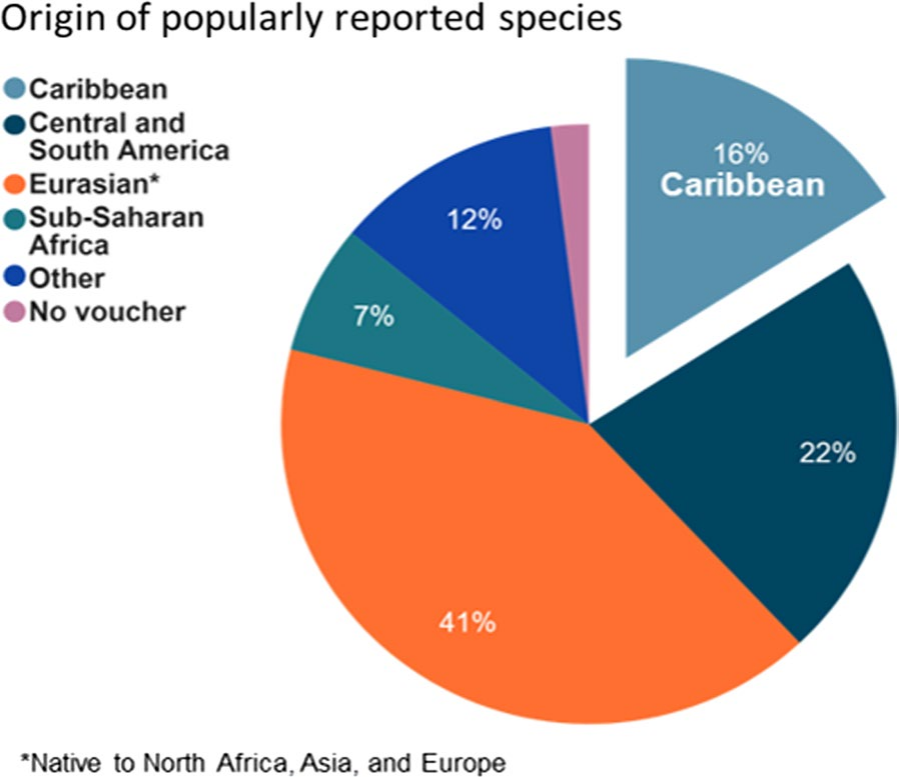
Geographic origin of popularly reported plants. Eurasia included Northern Africa, Asia, and Europe. Other locations included cultivated and/or globally distributed species

The majority of popular plants for women’s health in the NYC Haitian community were not native to Haiti (82%). More than half of the popular plant species in the Haitian community were also food plants (60%), such as *Abelmoschus esculentus* (L.) Moench (*kalalou*, okra), *Beta vulgaris* L. (*bètrav*, beets), *Daucus carota* L. (*kawòt*, carrots), or *Spinacia oleracea* L. (*epina*, spinach). These are primarily native to Eurasia (including North Africa, Asia, and Europe) and South and Central America (Fig. 3). Of the popularly used taxa native to Haiti, the majority consist of non-food plants employed only for medicinal purposes (83%).

### Comparison of women’s health concerns in NYC Haitian and Dominican communities

For the top six health conditions, more than half (> 60%) of Haitian women surveyed listed at least one use report for each of these conditions (Table 3). Almost all Haitian participants knew at least one remedy for birth and puerperium (97%) and gynecological infections (93%). The NYC Haitian community generally did not distinguish between specific types of gynecological infections, which are therefore groped together in Additional files 1 and 2. Dysmenorrhea (*χ*^2^ = 0.13; *p* = 0.72), infertility (*χ*^2^ = 0.42; *p* = 0.52), and contraception (*χ*^2^ = 0.51; *p* = 0.47) were similarly important in both the NYC Haitian and Dominican communities. However, Haitian women reported using plants for several conditions more frequently than Dominicans, including birth and puerperium (*χ*^2^ = 9.3; *p* = 0.002), gynecological infections (*χ*^2^ = 3.9; *p* = 0.05), vaginal cleansing (*χ*^2^ = 38; *p* =  < 0.0001), menstruation (*χ*^2^ = 30; *p* =  < 0.0001), and ovarian cysts/fibroids (*χ*^2^ = 29; *p* =  < 0.0001). For gynecological infections, Haitian participants frequently applied plants topically through washes, steams, douches, and baths (Table 4). The top five plants listed by Haitian participants for gynecological infections were *Cajanus cajan* (*pwa kongo*, pigeon pea; 131 use reports), *Cecropia peltata* L. (*fèy twonpé﻿t,* trumpet tree; 46 use reports), *Mentha spicata* L. and other *Mentha* spp. (*ti bonm,* spearmint; 123 use reports)*, Ricinus communis* L. (*maskreti,* castor; 42 use reports), and *Syzygium aromaticum* (*jiwòf*, cloves; 41 use reports) (Additional files 1 and 2).

**Table 3:** Comparison of top-reported women’s health conditions by NYC Haitian and Dominican communities based on plant–use reports (% of women listing at least one herbal remedy for each condition). Data from the NYC Dominican community were based on an earlier survey from 2005–2006, including 33 men and 93 women [30, 31, 37]

Women’s health condition	Percentage of NYC Haitian women reporting (*n* = 100 women)	Percentage of NYC Dominican participants reporting (*n* = 126 men and women)
Birth and puerperium#	97	47
Gynecological infections#	93	53
Vaginal cleansing#	69	10
Women’s health preventative*#	69	0
Dysmenorrhea	66	49
Pregnancy#	63	0
Hormonal imbalance#	47	0
Cancer prevention/treatment**#	45	4
Menstruation#	52	7
Infertility	40	37
Contraception	38	25
Abortion#	45	21
AIDS#	0	4
Gynecological cysts/fibroids#	46	5

*Preparation taking regularly for the purpose of maintaining reproductive health

**Participants listed medicinal plants for cancer generally as “cancer” as well as specifically for breast cancer

#Statistical significance between percentage of NYC Haitians reporting compared to NYC Dominicans (*p* ≤ 0.05)

**Table 4:** Methods to prepare plants for gynecological infections reported by NYC Haitians (*n* = 100 women for a total of 2484 use reports)

Method of treatment	Number of use reports by Haitian women
Intimate wash	504
Tea/beverage	491
Steam	70
Douche	46
Vaginal suppository	39
Bath	37
Topical application	6
Consumption	4
Massage	2
Unspecified	27

Haitian participants also frequently used plants as women’s health preventatives. These plant remedies were typically homemade and prepared as teas and taken daily, either alone or with other plants, for the purpose of maintaining reproductive health and preventing disease. For example, participants make a tea from the leaves of *Momordica charantia* L. (*asosi*, bitter melon) or *Mentha spicata* (*ti bonm,* spearmint) and drink a cup daily. Among the nine commonly mentioned preventatives for women’s health, five were identified as food plants. These were either brewed as tea or incorporated into daily consumption with the intention of promoting reproductive health.

### Medicinal plant use for women’s health in NYC Haitian and Dominican communities

**Fig. 4:**
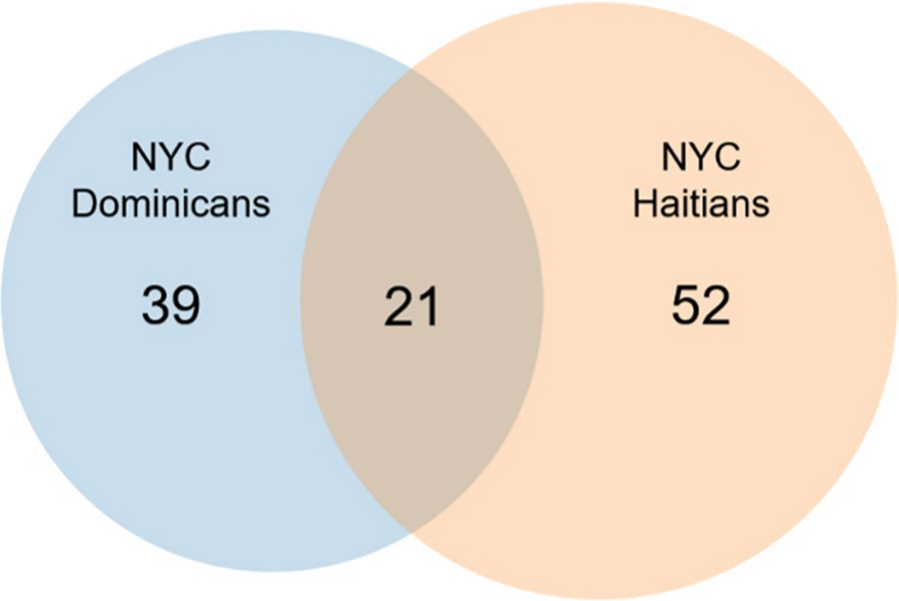
Venn diagram comparison of plant species used for women’s health reported independently by at least three participants in NYC Haitian survey (*n* = 100 women; orange circle) and NYC Dominican survey (*n* = 126 men and women; blue circle)

Compared to NYC Dominicans, among the 73 plant species independently mentioned by at least three Haitian women in NYC, 52 were exclusive to the Haitian community (Additional file 2). For one popularly used Haitian ethnotaxon, a tree bark with the local name *kòs bwa*, we were unable to obtain a voucher specimen and thus it remains unidentified. NYC Haitians shared 21 overlapping species with NYC Dominicans (JSI = 0.16) that are used for women’s health (Fig. 4). Of the 21 overlapping species, 38% are food plants, including *Allium sativum* L. (*lay,* garlic), *Beta vulgaris*, *Cajanus cajan* (*pwa kongo,* pigeon pea), *Cinnamomum* spp. (*kanél*, cinnamon), *Cocos nucifera* L. (*kokoye*, coconut), *Daucus carota* (*kawòt*, carrot), *Persea americana* Mill. (*zaboka*, avocado), and *Zingiber officinale* Roscoe (*jenjanm*, ginger) (Additional file 1). Comparatively, the percentage of popular food plants used for women’s health in the NYC Haitian community was notably higher than in the NYC Dominican community (NYC Haitians: 60%; NYC Dominicans: 32%; *z* = 3.3; *p* = 0.001).

Furthermore, although there are shared plants (foods and non-foods) used for women’s health between the two communities, very species few were used for the same purpose. For example, *Persea americana* (*zaboka*, avocado) is commonly used for birth and puerperium in the NYC Haitian community, but for abortion and contraception in the NYC Dominican community. Only four of the plants shared by the two communities (19%) have overlapping use for the same women’s health condition. Thus, *Aloe vera* (L.) Burm.f. (*lalwa*, aloe) is used by both groups for gynecological infections. Two other plant species, *Ricinus communis* (*maskreti*, castor) and *Zingiber officinale* (*jenjanm*, ginger), are used similarly for birth and puerperium, and one, *Cinnamomum* spp. (*kanél*, cinnamon), is shared for dysmenorrhea.

## Discussion

Our survey underscores the ongoing significance of medicinal plants for Haitian women’s health after migration to NYC and reveals the extensive knowledge of plant remedies for women’s health. Most participants interviewed reported knowing about and using plants for their health both before (97%) and after migration (83%). Furthermore, for the top six women’s health conditions, more than half of all participants knew at least one medicinal plant use. While this study had a specific focus on women’s health, 73 plant species were reported independently by at least three Haitian women. We also found that factors such as age, time living in the USA, and time living outside of Haiti did not impact women’s knowledge of medicinal plants. The rich knowledge of medicinal plants in the Haitian community for women’s health, both in terms of quantity of plant species and use reports, shows the wealth of information that could be explored in future ethnobotanical studies with a broader health focus.

Almost every Haitian woman interviewed knew at least one plant that was used for birth and puerperium. In Haiti, two-thirds of people live in rural areas of Haiti without immediate access to healthcare resources and nine out of ten births occur at home instead of a hospital and are attended by traditional birth attendants or family members [38]. In 2017, there were estimated 480 maternal deaths per 100,000 live births in Haiti [39]. Haitians and other Black women experience disparities in maternal and infant health in the USA as well [40, 41]. In 2021, the maternal mortality rate in the USA for non-Hispanic Black women was 69.9 deaths per 100,000 live births. While maternal mortality is considerably lower than in Haiti, these numbers are still higher than the maternal mortality for non-Hispanic White women in the USA: 26.6 deaths per 100,000 live births [42]. The Haitian community reported significantly more uses of plants for labor, puerperium, and pregnancy than previously in the NYC Dominican community. The US Dominican community also experiences inequality in maternal and infant health [40, 43]. In 2021, the maternal mortality rate for Hispanic women was 28 deaths per 100,000 live births. However, lack of access to healthcare for Haitian women, both before and after migration, has made medicinal plants for childbirth and pregnancy important to recovery [38, 44]. The cultural importance of medicinal plants for childbirth and pregnancy, along with the medicinal plants used, is carried across borders to the Haitian community in NYC due to the risk experienced when women were living in Haiti as well as new barriers to healthcare after moving to the USA, such as language, cost, and access to insurance [13].

Gynecological infections, including sexually transmitted infections (STI) and vaginal infections, were also a top condition for NYC Haitians that nearly every woman knew how to treat with medicinal plants. In the Dominican community, gynecological infections were also a top women’s health condition treated with medicinal plants; however, there were differences in how the uses were reported. NYC Dominicans classified vaginal infections by symptoms, such as *flujo vaginal* (vaginal discharge) and *comezón vaginal* (vaginal itching), and mentioned specific STIs by name [45]. The NYC Dominican community also frequently uses complex plant mixtures extracted in alcohol (*botellas*) or boiled for an extensive time in water (*bebedizos*) to treat reproductive and genitourinary conditions, including gynecological infections. *Botellas* used by Dominicans for genitourinary conditions are typically taken internally, and *bebedizos* are consumed during puerperium to cleanse the uterus after childbirth [29]. The NYC Haitian community most frequently reported extracting plants in water and using them as an intimate wash to treat gynecological infections. Understanding cultural differences in how groups of people treat, understand, and interpret disease is crucial information for medical professionals who have patients of different cultures [46]. Physicians also need to know for which health conditions people preferentially use medicinal plants, and what these plants are through taxonomic authentication [34].

Vaginal cleansing was another treatment that had cultural importance for Haitian women. The use of medicinal plants and other natural and/or synthetic agents to clean, wash, and tighten the vagina is common in Haitian and other cultures with African descent [47]. Vaginal cleansing is associated with an increased risk of vaginal and STI infections, such as bacterial vaginosis, HIV, and human papillomavirus [26, 48]. Increased risk is linked with inflammation, stripping of the protective vaginal mucosa, as well as the disruption of the vaginal microbiota [49, 50]. When used intravaginally, compounds from plants and other substances, like permanganate, come in direct contact with the vaginal microbiota. The vaginal microbiota is primarily composed of beneficial *Lactobacillus* bacteria that naturally produce lactic acid and bacteriocins that protect against infection. When the microbiota is disrupted, women are more susceptible to recurrent and more severe infections [51]. Plants used for cleansing are not the only treatment applied intravaginally. Both NYC Haitians and Dominicans [45] frequently apply plants for gynecological infections directly to the vagina as washes, douches, or steams. There has been very little ethnopharmacological research into the effect of medicinal plants on the beneficial vaginal bacteria. We previously identified plants used for gynecological infections and vaginal cleansing that were used by the Dominican community in NYC and likely important to the Haitian community [45]. This survey has identified the top five plant species used to treat gynecological infections that warrant further investigation for their effect on the vaginal microbiota: *Cajanus cajan, Cecropia peltata*, *Mentha* spp.*, Ricinus communis*, and *Syzygium aromaticum.*

The Haitian survey instrument included specific questions about plants used as women’s health preventatives, hormonal imbalances, and gynecological cancers that were not included explicitly in the Dominican survey, which may have influenced the frequency of reports in the intercultural comparison. Women’s health preventatives were defined as plants not used for a specific illness but instead to support overall wellness for reproductive health. The number of use reports for these preparations shows the importance of preventative medicine for Haitian women. There were also women’s health conditions participants reported that could not easily be self-diagnosed, such as gynecological cysts/fibroids and cancers. However, Haitian participants mentioned frequently in interviews that they would go to the doctor to receive a diagnosis and then self-treat or seek the help of a healer to treat the condition with medicinal plants. Furthermore, when participants reported using plants for cancer, it was typically reported as cancer prevention. *Dysphania ambrosioides* (L.) Mosyakin & Clemants (*simen kontra*, Mexican tea) was frequently taken as a daily tea for cancer prevention because it is believed to be “good for everything.” Similarly, the fruit of *Annona muricata* L. (*korosol*, soursop) was eaten daily to prevent cancer. Both plants have research supporting their potential for cancer preventative properties. *Dysphania ambrosioides* seed extracts inhibited the invasion and migration of SMMC-7221 cells in hepatocellular carcinoma [52]. *Annona muricata* has been widely studied for anticancer activity, including in vitro cytotoxic, antiproliferative, and other cancer preventative activity, such as upregulation of apoptotic genes. It also showed anticancer activity in vivo activity in rats for liver and prostate cancer [53]. The importance of preventative medicine for women’s health, particularly the regular consumption of “healthy” food plants or teas, highlights the role food plants play in maintaining health without seeking treatment for a particular condition [54].

Food plants made up the majority of the Haitian plant inventory for women’s health, and more than one-third of the plant species that overlapped among NYC Haitians and NYC Dominicans were food plants. This shows that the Haitian women’s health pharmacopeia in NYC is shaped by commonly available plants that are global in origin and well-established across the Caribbean region in the foodways of Afrodescendant communities [55]. Traditional knowledge about food plants is often non-proprietary and thus easily exchanged between different communities living in close geographic proximity, either in their birth and/or host countries, as it appears to be the case in our study between members with a shared Afrodescendant heritage [31]. Moreover, for Caribbean communities, foods became even more important as medicines during the COVID-19 pandemic [54]. Together, these results show: (1) the dynamic character of this subset of traditional knowledge and that (2) ethnobotanists should not treat all traditional knowledge as a uniform block retraceable in its entirety to deep historical roots; instead some aspects of traditional knowledge systems may be more contemporary in origin and highly dynamic in character, the latter both in terms of the plant species used, as well as the adaptability of their uses for new and emerging health conditions. In this aspect, food plants represent an ever available, relatable, culturally identifiable, and highly adaptable component of contemporary plant knowledge that can be swiftly exchanged in-person and/or on social media when the need arises.

Intracultural comparisons between Dominicans living in the DR and NYC found that fewer non-food plant species (plants used solely as medicines without culinary applications) are available after migration and are instead replaced with plants readily available in markets and grocery stores [30, 31]. While food plants were used for a wide range of women’s health conditions in the Haitian community, participants also emphasized the importance of “eating well” to maintain their health particularly during pregnancy. Specific species such as beets, carrots, and spinach were listed, as well as leafy greens and vegetables in general, particularly those that are high in iron. Anemia is a major public health concern in Haiti, particularly for pregnant and lactating women [56]. Five participants free-listed anemia as a women’s health issue that they treat with medicinal plants. This is consistent with previous ethnobotanical research with women in Haiti that highlighted the importance of self-treating anemia with food plants that are high in iron [57].

Our survey showed there exists a strong network in NYC to source plants directly from Haiti. More than three quarters of study participants who use plants in NYC reported buying medicinal plants from NYC Haitian vendors, directly from Haiti, or from Haitian community members in NYC. This result aligns with previous literature reporting on the dissemination of Haitian plants in the Canadian and US diaspora. Medicinal plants from Haiti are central and significant for preserving culture in Haitian diaspora communities and therefore, Haitian business owners and community members ensure that culturally important Haitian medicinal plants are widely available where the diaspora is located [8]. In contrast, the NYC Dominican community reported primarily procuring plants from *botánicas,* which serve as spiritual and healing centers for different, but often Spanish-speaking, Caribbean communities in the city [31, 58]. This result highlights the intricacy of the herbal commerce system in NYC—where even groups of people from the same island in the Caribbean, such as Haitians and Dominicans from Hispaniola, buy and source their plants in distinct ways.

We acknowledge some limitations of our recruitment methods and study design. Haitian participants who were interviewed had self-reported familiarity with plants. However, during the recruitment screening process, every woman we approached knew about medicinal plants. Therefore, this recruitment strategy likely did not influence the randomness of our sample. However, many of the interviews were conducted at Haitian Americans United for Progress (HAUP) offices in Brooklyn and Queens. HAUP provides social services for the NYC Haitian community, particularly for people who have recently moved to the USA. This recruitment strategy may have influenced representation in the sample population. Finally, contrary to our Haitian survey which was only based on the knowledge, beliefs and practices of women, the Dominican survey also included use reports from male participants about women’s health. Nevertheless, in the latter survey, there were no obvious differences between men and women in the kinds of plants or conditions reported.

We developed our hypotheses for this work through review of the literature and previously collected data [45]. The first hypothesis, that Haitians in NYC would have significant plant knowledge for women’s health, was supported by the results of this paper. However, more differences existed in the plants and women’s health conditions treated with medicinal plants between the NYC Dominican and Haitian communities than previously hypothesized. This research was initially developed during the COVID-19 pandemic to continue research in a time when an in-person survey was not possible. In the wake of the pandemic, these results highlight the importance of conducting field ethnobotanical survey work to ground-truth similarities and differences between communities. These results could not have been predicted by literature or previous work with related communities in NYC. In-person interviews with participants are crucial to understanding plant knowledge in a community.

## Conclusion

Results from our ethnomedical survey demonstrated the continuity of medicinal plant use for women’s health in NYC Haitian women through self-reported use, consistency in extensive plant knowledge across community members, and an existing network of commerce for Haitian plants in NYC. Popular plants in the Haitian community for women’s health reported independently by at least three women were easily accessible global food plants that also have medicinal uses, including esteemed Caribbean foods such as *Cajanus cajan* and *Zingiber officinale*. The Haitian plant pharmacopeia for women’s health is known by Haitian women of all ages, regardless of their time living in NYC, and is inherently Caribbean but at the same time global in terms of the geographic origin of its plants and their ease of procurement away from home. It thus represents a resource for community care that is easily shared and adaptable in response to the challenges and changing health needs of urban immigrant life. Cross-cultural comparisons with the Dominican community revealed similarities, but also culture-specific differences in plants used and women’s health conditions prevalently treated with medicinal plants, particularly for birth and puerperium, gynecological infections, vaginal cleansing, and ovarian cysts/fibroids. In the Haitian community, the importance of treating certain women’s health conditions with medicinal plants, such as birth and puerperium, may reflect the high maternal mortality rate. The ethnobotany of women’s health is a topic that remains vastly under-represented in the literature compared to its global importance. Future collaborations with health clinics in NYC might expand on this topic with the shared goal to improve health care to underserved Haitian women. Cross-cultural comparisons of plant knowledge between related communities are important for recognizing culturally specific public health needs. Furthermore, the result of this comparison could not be predicted by previously collected data or the existing literature—underlining the importance of ground-truthing ethnobotanical research within communities.

### Supplementary Information

**Additional file 1.** Plant species shared by NYC Haitian and NYC Dominican Communities and top reported conditions by participants (for conditions mentioned five times or more for any plant species). Scientific names verified by Catalogue of Life (https://www.catalogueoflife.org/col/details/database/id/24). * Indicates shared plant use with NYC Haitian Community. # Indicates data is from Vandebroek and Balick, 2012. Number of use reports for top-reported conditions shown by numbers in paratheses.

**Additional file 2.** Unique plant species reported by NYC Haitians and top reported conditions by participants (for conditions mentioned five times or more for any plant species). Scientific names verified by Catalogue of Life (https://www.catalogueoflife.org/col/details/database/id/24). Number of use reports for top-reported conditions shown by numbers in paratheses.

## Data Availability

Data will be made available upon request.

## Abbreviations

DR: Dominican Republic
HAUP: Haitian Americans United for Progress
JSI: Jaccard similarity index
NYBG: New York Botanical Garden
NYC: New York City
USA: United States of America
NY: William & Lynda Steere Herbarium
